# An Unusual Case of Escherichia Coli Meningitis in an Immunocompetent Adult

**DOI:** 10.7759/cureus.37954

**Published:** 2023-04-21

**Authors:** Mikael Mir, Esraa Hassan, Ahmed Sharaf, Abbas B Jama, Sydney Boike, Ibtisam Rauf, Noura Attallah, Cristina Corsini Campioli, Eric Gomez Urena, Syed Anjum Khan

**Affiliations:** 1 Medical Student, University of Minnesota Medical School, Minneapolis, USA; 2 Critical Care Medicine, Mayo Clinic Health System, Mankato, USA; 3 Internal Medicine, Baptist Hospital of Southeast Texas, Beaumont, USA; 4 Medical Student, St. George’s School of Medicine, University Centre Grenada, St. George’s, GRD; 5 Internal Medicine, Mayo Clinic, Rochester, USA; 6 Infectious Disease, Mayo Clinic Health System, Mankato, USA

**Keywords:** e. coli bacteremia, e. coli, gram-negative meningitis, bacterial meningitis, lumbar puncture, meningitis, cerebrospinal fluid, gram-negative bacillus, escherichia coli

## Abstract

Spontaneous meningitis caused by Gram-negative bacilli is rare in adults. It typically occurs after a neurosurgical procedure or head injury but may also be related to the presence of a neurosurgical device, cerebrospinal fluid (CSF) leak syndrome, or seen in immunosuppressed patients. *Escherichia coli* (*E. coli*) is the leading cause of Gram-negative bacilli meningitis. We describe the case of a 47-year-old man who was hospitalized for spontaneous, community-acquired *E. coli* meningitis, which is unusual to see in an immunocompetent adult. CSF analysis was consistent with bacterial meningitis; his blood culture was positive for *E. coli.* Within 24 hours of initiation of antibiotics, his status improved.

## Introduction

Bacterial meningitis is a serious infection of the meninges and can be caused by a variety of bacteria, with the most common pathogens including *Streptococcus pneumoniae*, *Neisseria meningitidis*, and *Haemophilus influenzae* [1]. In this case report, we present an unusual case of meningitis caused by *Escherichia coli* (*E. coli*) in an adult, a common bacterium usually associated with gastrointestinal and genitourinary infections. While *E. coli* is known to cause meningitis in neonates, it is a rare occurrence in adults [2]. This case highlights the importance of considering atypical causes of meningitis in patients who present with typical symptoms of the disease, even in the absence of traditional risk factors.

## Case presentation

A 47-year-old male with a past medical history significant for diabetes mellitus with haemoglobin A1c of 7.0 and hypertension presented to the emergency department (ED) for progressive altered mental status via ambulance. The patient began complaining to his wife about headaches, generalized body aches, and chills around noon that day. He had no cough, runny nose, or urinary symptoms. Late in the afternoon, the patient was not verbally responsive or able to follow commands. On arrival at the ED, the patient continued to have altered mental status with fever (T max 39.3°C). A review of systems found the patient positive for lethargy, chills, diarrhea, weakness, headache, and confusion. On exam, he was noted to be febrile with a tympanic temperature of 39.3°C, heart rate of 104 beats per minute, respiratory rate of 24 breaths per minute, blood pressure of 150/90 mmHg, and fingerstick glucose of 249 mg/dL. The patient appeared lethargic, agitated, nonverbal, and uncooperative. Glasgow Coma Scale (GCS) score was eight. Neck rigidity was present. No rash was noted. Peripheral pulses were bounding with a good capillary refill. Cardiac, lung, and abdominal exams were unremarkable. The neurological exam was non-focal. Pertinent infectious diseases history includes eating ring bologna, a finely ground pork sausage made in the form of a ring, 24 hours before the presentation that was sitting on the counter for two days.

**Figure 1:**
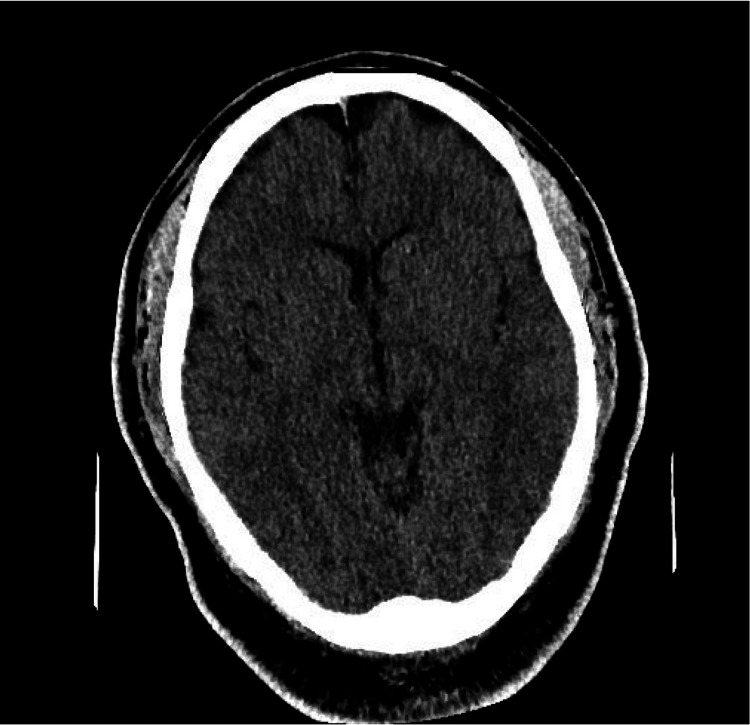
CT of the head with unremarkable findings

Blood cultures and routine laboratory tests were obtained, and fluid resuscitation as per severe sepsis protocol was initiated. Piperacillin-tazobactam and vancomycin were given for sepsis of unclear source. Computed tomography (CT) of the head, with and without contrast, was negative for any acute pathology (Figure 1). A lumbar puncture was performed after antibiotics were administered. CSF was noted to be turbid. His antimicrobial regimen was adjusted to ceftriaxone, ampicillin, vancomycin, and acyclovir. Laboratory results are shown in Tables 1-2.

**Table 1:** Laboratory Results L = liter; g/dL = grams per deciliter; mmol/L = millimoles per liter; mg/dL = milligrams per deciliter

Basic Labs	Results	Reference Range
White Blood Cells	11.5	3.4-9.6x10^9^/L
Hemoglobin	14.4	12.2-16.6 g/dL
Hematocrit	41.0	38.3-48.6%
Platelets	172	135-317x10^9^/L
Sodium	127	135-145 mmol/L
Chloride	90	98-107 mmol/L
Blood Urea Nitrogen	10	8-24 mg/dL
Creatinine	0.77	0.74-1.35 mg/dL
Calcium	8.3	8.6-10.0 mg/dL

**Table 2:** CSF Analysis CSF = cerebrospinal fluid; mmH_^2^_O = millimeters of water column; mg/dl = milligrams per deciliter

CSF Findings	Results	Normal
Gross Appearance	Slightly cloudy	Clear
Pressure	Elevated	<180 mmH_2_O
Total Nucleated Cells	195	Adults 0-5
Neutrophils	85	Adults 2-4%
Protein	348	15-45 mg/dl
Glucose	123	45-100 mg/dl
CSF to serum ratio	0.48	>0.4
Gram Stain	Negative	Negative

Blood cultures showed growth of *E. coli* within eight hours of incubation. CSF bacterial cultures, meningitis/encephalitis PCR panel (including for *E. coli* K1), and the Strongyloides antibody were all negative (Table 3). Within 24 hours of antimicrobial therapy, his mental status started to improve.

**Table 3:** Bacterial, viral, and fungal panel results from CSF

	Latest Reference Range & Units	01/05/21 10:08	07/30/21 13:01	11/27/22 18:05	11/27/22 18:19	11/27/22 19:53	11/29/22 05:10
Escherichia coli K1	Negative					Negative	
Haemophilus influenzae	Negative					Negative	
Listeria monocytogenes	Negative					Negative	
Neisseria meningitides	Negative					Negative	
Streptococcus agalactiae	Negative					Negative	
Streptococcus pneumoniae	Negative					Negative	
Cytomegalovirus	Negative					Negative	
Enterovirus	Negative					Negative	
Herpes Simplex Virus 1	Negative					Negative	
Herpes Simplex Virus 2	Negative					Negative	
Human Herpes Virus 6	Negative					Negative	
Human Parechovirus	Negative					Negative	
Varicella Zoster Virus	Negative					Negative	
Cryptococcus neoformans/gattii	Negative					Negative	
Strongyloides Ab, IgG, S	Negative						Negative
Enterovirus PCR	Negative					Negative	
HSV 1 PCR, C	Negative					Negative	
HSV 2 PCR, C	Negative					Negative	
Influenza A, POCT	Negative				Negative		
Influenza B, POCT	Negative				Negative		
Resp Syncytial Virus, POCT	Negative				Negative		
Varicella-Zoster Virus PCR	Negative					Negative	
SARS CoV-2 RNA, TMA	Undetected	Undetected	Undetected				
SARS CoV-2, PCR, Rapid, V	Undetected			Undetected			

The isolate of *E. coli* was susceptible to ceftriaxone; hence the patient completed a 21-day course of ceftriaxone intravenously.

## Discussion

*E. coli* is one of the most frequent organisms that cause meningitis in newborns. However, it is highly uncommon for immunocompetent adults to develop community-acquired *E. coli* meningitis, with a prevalence of one case annually documented worldwide since 1945 [3]. In contrast, *Pseudomonas aeruginosa*, *Klebsiella pneumoniae*, and *Acinetobacter* species are the most prevalent Gram-negative bacilli that cause bacterial meningitis in adult patients [4]. Therefore, community-acquired *E. coli* meningitis in immunocompetent adults is a rare occurrence, and the factors leading to this presentation should be considered for future cases.

Most cases of meningitis caused by *E. coli* have been healthcare-associated infections following head injuries or neurosurgical procedures [5]. However, spontaneous *E. coli* meningitis can occur in patients with several comorbidities [6], including chronic alcoholism, cirrhosis, human immunodeficiency virus infection (HIV), chronic obstructive lung disease, immunosuppressive medications, and diabetes mellitus [7]. Strongyloides hyperinfection syndrome can present with *E. coli* meningitis as the Strongyloides larva migrates from the gastrointestinal mucosa to disseminate through the body carrying *E. coli* in the process [8]. In our patient, the Strongyloides antibody test was negative.

Our patient presented with a headache, fever, and altered mental status. These symptoms, along with neck stiffness, raise concern for meningitis. CSF analysis showed pleocytosis with neutrophilia and elevated protein with a borderline low CSF to serum glucose ratio, which was suggestive of bacterial meningitis. The CSF Gram stain was negative; however, this is not a sensitive test to diagnose bacterial meningitis. The bacterial cultures from CSF were negative, but unfortunately, a lumbar puncture was performed after administering antibiotics (piperacillin-tazobactam). Several studies have shown that the administration of antimicrobial therapy prior to the collection of CSF samples can decrease the sensitivity of the CSF culture results [9]. In pediatric populations, antibiotics sterilize the CSF fluid as early as less than one hour after antibiotic administration [9]. Therefore, the positive blood cultures for *E. coli*, clinical presentation, and CSF findings suggest *E. coli* meningitis. The meningitis PCR panel in our patient was negative for *E. coli* K1, and *E. coli* carrying the K1 capsule antigen is responsible for 77% of neonatal *E. coli* meningitis cases. It is possible that this infection was due to an *E. coli* without the K1 antigen.

Pomar et al. [10] found that patients with Gram-negative bacilli meningitis had a death rate of 53%. In a different study, Bodilsen et al. reported a mortality rate of 36% among patients with *E. coli* meningitis, which is higher than 20% mortality with pneumococcal meningitis and 7% mortality with meningococcal meningitis [6]. This difference in Gram-negative bacilli meningitis between the studies may be related to the specific Gram-negative bacillus causing the infection and specific virulence traits of the strains responsible for the infection [11]. Although *E. coli* meningitis can result in several complications, sepsis and multi-organ failure are the most common causes of death in these cases. In our patient, the clinical course was standard due to the fast clinical improvement with antimicrobial therapy. However, this may be related to the virulence of this strain of *E. coli*. If the strain of *E. coli* in our patient had the K1 capsule antigen or other virulence factors, the patient might have had additional complications.

## Conclusions

In conclusion, *E. coli* meningitis in immunocompetent adults is unusual. As seen in our patient, risk factors often include diabetes mellitus but are not limited to cirrhosis, HIV infection, chronic obstructive pulmonary disease, and chronic organ insufficiency. The overall prognosis depends on awareness of the disease and how quickly antibiotics are administered. Impaired consciousness or bacteremia are worse prognostic factors. Given the rare prevalence of this case, the risk factors leading to this presentation are important to consider for future patients.
